# Physical, Physiological, and Technical Demands in Ultimate Frisbee Small-Sided Games: Influence of Pitch Size

**DOI:** 10.3390/sports9080104

**Published:** 2021-07-23

**Authors:** Masanobu Kajiki, Yuto Yamashita, Ryosuke Inada, Takaaki Matsumoto

**Affiliations:** Laboratory for Exercise Physiology and Biomechanics, Graduate School of Health and Sport Sciences, Chukyo University, 101 Tokodachi, Kaizu-cho, Toyota 470-0393, Japan; yutoyama0711@gmail.com (Y.Y.); inada.ss17@gmail.com (R.I.); t-mat@sass.chukyo-u.ac.jp (T.M.)

**Keywords:** heart rate, blood lactate concentration, global positioning system, flying disc

## Abstract

Small-sided games (SSGs) are common drills used in various team sports, but the exercise intensity in ultimate Frisbee SSG has not yet been investigated. To clarify the physical, physiological, and technical demands of ultimate Frisbee SSG, we investigated the influence of pitch size on exercise intensity during SSG. Nine male college ultimate Frisbee players played (3 vs. 3) SSG on small (SSG_S_: 30 × 15 m) and large (SSG_L_: 40 × 20 m) pitches; both SSGs comprised of four 4 min periods, interspersed by 5 min of passive recovery. Players’ mean heart rate (170 ± 8 and 171 ± 7 bpm), peak heart rate (184 ± 7 and 184 ± 5 bpm), and blood lactate concentration (11.3 ± 4.7 and 11.8 ± 4.6 mmol/L) were similar in SSG_S_ and SSG_L_, respectively. The total distance covered (1984 ± 166 m and 1702 ± 80 m) and the distance covered during quick (860 ± 112 m and 696 ± 69 m) and high-intensity running (439 ± 95 and 255 ± 44) in SSG_L_ were significantly longer than those in SSG_S_ (*p* < 0.05). Conversely, the number of accelerations (45 ± 3 and 41 ± 3) and decelerations (44 ± 3 and 40 ± 4), catching errors (2 ± 1 and 1 ± 1), and turnovers (8 ± 2 and 6 ± 2) in SSG_S_ were significantly greater than those in SSG_L_ (*p* < 0.05). This study suggests that ultimate Frisbee SSG provides high-intensity training, which stimulates the glycolytic pathway. Furthermore, manipulating SSG pitch size effectively modulates the physical demands of SSG.

## 1. Introduction

Ultimate Frisbee is a noncontact, team-based sport played by millions of people across approximately 50 countries [1,2]. Sex-specific or mixed-sex games are played either outdoor or indoor, with five to seven players per team [1,2,3,4]. Official matches are played on a 100 m long × 37 m wide pitch, with end zones (18 m × 37 m) at either end of the pitch [5]. Players throw a disc and aim to score goals by catching it in the attacking end zone. However, as a pivot location must be established (the toe of one foot must remain planted in one spot) immediately after receiving a pass, the player possessing the disc can pivot but cannot travel (no displacement from that fixed position can occur [5]) until they have thrown the disc. Therefore, disc throwing skill as well as the movement of players when they do not possess the disc are important for scoring goals. Players repeatedly perform high-intensity movements, such as sprinting and changing direction throughout the game, in order to receive an effective pass when playing offense, and to prevent losing points when playing defense.

The physical and physiological demands on players during ultimate Frisbee games are considered to be comparable with those in various intermittent team sports, such as soccer [6] and rugby [7]. A previous study showed that collegiate male ultimate Frisbee players covered 4.7 ± 0.5 km, of which high-intensity running (14–22 km/h) and sprinting (>22 km/h) accounted for 0.6 ± 0.1 km and 0.2 ± 0.1 km, respectively, in a 54 min game [4]. The same study also reported that mean and peak heart rate (HR) during the ultimate Frisbee game reached 160 ± 6 bpm (82 ± 2% of each player’s maximum HR) and 192 ± 6 bpm (99 ± 1% of each player’s maximum HR), respectively [4]. Another study showed that during a 36 min ultimate Frisbee game, played by recreational male players (5 vs. 5), mean HR, peak HR, and blood lactate concentration (BLa) were 172 ± 12 bpm (88 ± 6% of maximum HR), 190 ± 14 bpm, and 5.5 ± 1.6 mmol/L, respectively [1]. In an official national-level match, peak sprinting velocity reportedly reached 26.0 ± 3.5 km/h [3].

Training drills that are conducted with a lower number of players and on a smaller area pitch than those used in regular games, known as small-sided games (SSGs), are commonly used to develop players’ endurance, agility, technique, and tactical abilities in team sports [8,9]. An understanding of the exercise intensity required during training drills, such as SSG, is believed to be important in order to enhance training effects and prevent overtraining; however, exercise intensity in ultimate Frisbee SSG has not been fully investigated. In ultimate Frisbee, as the player possessing the flying disc cannot travel, only players not possessing the disc repeatedly perform intense movements, such as sprinting, and changing direction. SSG training can be used to elicit more intense and complex set plays by increasing the frequency of participation in attacks of the players not possessing the disc. In ultimate Frisbee, exercise intensity during SSG might be higher than that during regular games; therefore, it is necessary to clarify the physical, physiological, and technical demands of ultimate Frisbee SSG on players.

The manipulation of variables, such as pitch size, number of players, and game duration can modulate exercise intensity in SSG in various team sports [8]. In particular, pitch size is the main factor that influences the training intensity of an SSG, and, therefore, is considered to be a useful variable to enhance training stimulus [8]. One study showed that in rugby SSG, total distance and the distances covered at moderate and high velocities were greater on a large pitch (40 m width × 70 m length) than those covered on a small pitch (10 m width × 40 m length) [10]. Furthermore, players’ HR and BLa have been reported to be greater in soccer SSG with an increased pitch size [11]. In addition, although the use of a format with four 4 min SSG is recommended to elicit high exercise intensity [8], technique deteriorates in repeated bouts and technical errors increase from accumulated fatigue [12,13]. Understanding the influences of pitch size and repeated bouts of SSG on the physical, physiological, and technical demands on players will be useful to optimize training effects.

The purpose of the present study was to clarify physical, physiological, and technical demands on players during ultimate Frisbee SSG. We investigated the influences of manipulating pitch size and playing multiple bouts (i.e., SSG periods) on the physical, physiological, and technical demands on players. We hypothesized that with a larger pitch size, distances covered (total distance and distances covered in high-intensity running and sprinting) would be greater, and that HR and BLa would be higher. Additionally, we hypothesized that technical errors would increase during repeated bouts of SSG.

## 2. Materials and Methods

### 2.1. Participants

Fifteen members of the Chukyo University Ultimate Frisbee Club volunteered to participate in this study. SSG sessions were conducted in a random order, with two pitch sizes (two each; therefore, four sessions in total), during the club’s preseason preparation phase (late February and March). Of the 15 players, 9 players played in SSG on both pitch sizes, and 6 players played SSG on only one pitch size; analysis was conducted using data from the 9 participants (mean ± standard deviation: height, 172 ± 5 cm; weight, 64 ± 7 kg; age, 21 ± 1 years) who participated in SSG sessions on both pitch sizes. All players were intercollege level athletes who trained 4 sessions per week for more than 2 h per session and had at least 1 year of ultimate Frisbee training (including SSG). All participants were notified of the research procedures and the potential benefits and risks, and appropriate written informed consent was obtained from all participants. The study was approved by the research ethics committee of Chukyo University in conformity with the Declaration of Helsinki (No. 2020-44).

### 2.2. Experimental Design

SSG took place on a natural lawn pitch at the same time of day (10:00–11:00 a.m.); each session was separated by at least 48 h. The small (SSG_S_, 30 × 15 m) and large (SSG_L_, 40 × 20 m) pitches both had length-to-width ratios of 2:1; the end zones were 7 × 15 m and 8 × 20 m, respectively. SSG sessions comprised four 4 min periods, interspersed by 5 min of passive recovery, played with three players per team (3 vs. 3). A previous study recommended the use of a format with four 4 min SSG, in order to elicit high exercise intensity [8]. In our study, the rest between periods was set to 5 min, to allow time to collect BLa. In each session, SSG were played after the same standardized 30 min warm-up, which comprised of static and dynamic stretching, low- and high-intensity running, and technical movements. The players were free to choose which offensive scheme they wished to use; however, in order to increase their exercise intensity, we asked all players to play man-to-man defense [14]. The same encouragement to maintain a high work rate was provided throughout all sessions, and all periods of SSG were initiated by a standard pull—a throw from a defensive player from the end zone [5]. After a point had been scored, play resumed from the end zone. The clock ran continuously throughout each game, and games were self-officiated [5].

### 2.3. Measures

HR was continuously recorded. Each player wore an HR monitor (Polar, Polar Electro Oy, Kempele, Finland) and global positioning system (GPS) unit throughout each SSG session. HR monitors were synchronized with the GPS units; the sampling rate was 18.18 Hz. Each player’s mean and peak HR were calculated for each period of SSG, and the relative value to age-predicted maximum HR (%HRmax) was calculated as follows [15]: %HRmax = 100 × (exercise HR/(220 – age)). This method is identical to that used in previous studies that examined HR responses during regular ultimate Frisbee games [1,2]. BLa was determined from a capillary blood sample that was drawn from the fingertip (Lactate Pro2, ARKRAY Inc., Kyoto, Japan) after each period. Rating of perceived exertion (RPE) was also recorded using the Borg scale (6, no exertion at all; 7, extremely light; 9, very light; 11, light; 13, somewhat hard; 15, hard; 17, very hard; 19, extremely hard; 20, maximal exertion) [16] immediately after each period.

Participants’ GPS units (GPexe Pro2, Exelio Srl, Udine, Italy) measured movement during SSG sessions. The GPS unit was placed on the back between the shoulders to obtain optimal satellite signals. Each GPS unit acquired data at 18.18 Hz, and the distance covered, speed, and acceleration were determined. The total distance covered, distance covered in five speed categories, number of accelerations and decelerations, and peak speed were calculated for each period of SSG. Five speed categories were established: walking (0–4 km/h), jogging (4–8 km/h), quick running (8–14 km/h), high-intensity running (14–22 km/h), and sprinting (>22 km/h). These speed categories are comparable with those used in a previous study, that examined physical demands during a competitive ultimate Frisbee game [4]. Acceleration and deceleration bouts were categorized as follows: high acceleration (>3 m/s^2^), moderate acceleration (2–3 m/s^2^), low acceleration (1–2 m/s^2^), low deceleration (−1 to −2 m/s^2^), moderate deceleration (−2 to −3 m/s^2^), and hard deceleration (<−3 m/s^2^) [17,18].

All SSGs were recorded using digital video cameras (GZ-R480, Jvc Kenwood, Kanagawa, Japan) that were positioned around the pitch area. A hand notation system was used to assess the technical actions and errors of each player during SSG. The number of passes (i.e., the number of throws of the flying disc), percentage of successful passes, number of catching errors by the receiver, number of turnovers in which each offensive player was involved, and number of interceptions made by each defensive player were counted for each period of the SSG by two researchers experienced in ultimate Frisbee. The test–retest reliability of this system was checked using intraclass correlation coefficient (ICC_3,1_) [19]. Test–retest reliability ICCs were 0.991 (95% confidence interval (CI): 0.986–0.995) for number of passes, 0.976 (95% CI: 0.962–0.981) for percentage of successful passes, 0.873 (95% CI: 0.804–0.918) for number of catching errors, 0.957 (95% CI: 0.932–0.973) for number of turnovers, and 0.848 (95% CI: 0.768–0.902) for number of intercepts.

### 2.4. Statistical Analyses

All data are presented as mean ± standard deviation. Data were compared using two-way repeated-measures analysis of variance (pitch size (SSG_S_ and SSG_L_) × period (1, 2, 3, and 4)). Because the BLa of some players could not be measured after periods 1 and 3, BLa was compared using only data from periods 2 and 4. Post hoc analyses were performed using Bonferroni correction when a significant main effect or interaction was detected. For post hoc analyses, Cohen’s *d* effect size (ES) was calculated and assessed as small (0.2–0.5), moderate (0.5–0.8), or large (>0.8) [20]. Statistical significance was set at *p* < 0.05. All statistical analyses were performed using statistical software (SPSS v26, IBM Corp., Armonk, NY, USA).

## 3. Results

**Figure 1:**
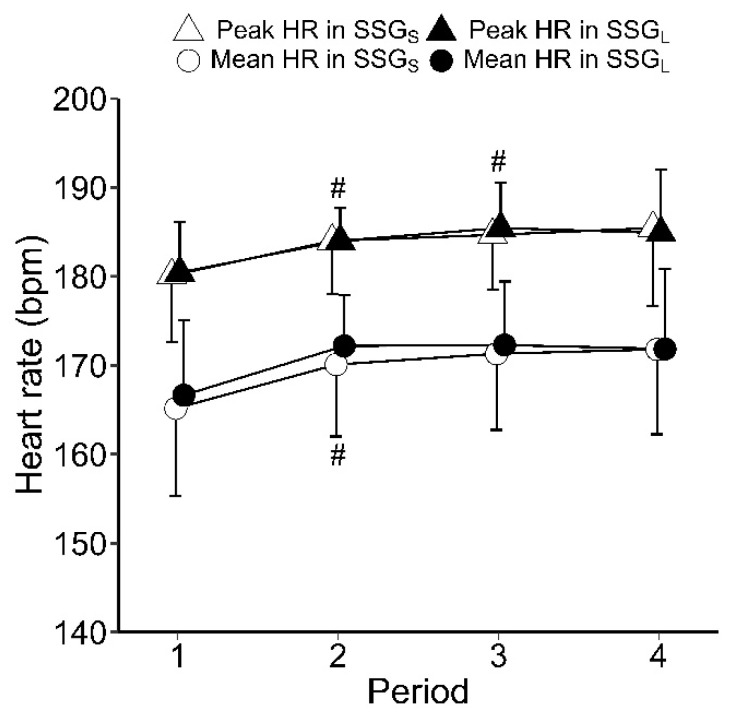
Mean and peak heart rate during each period in SSG_S_ and SSG_L_. # Significant difference (Corrected-*p* < 0.05) compared with period 1. Values are presented as mean ± standard deviation.

The pitch size × period interaction was not significant for mean HR, peak HR, BLa, or RPE. Overall, mean HR was 170 ± 8 bpm (85 ± 4 %HRmax) in SSG_S_ sessions and 171 ± 7 bpm (86 ± 4 %HRmax) in SSG_L_ sessions; peak HR reached 184 ± 7 bpm (92 ± 4 %HRmax) in SSG_S_ sessions and 184 ± 5 bpm (92 ± 3 %HRmax) in SSG_L_ sessions. Mean and peak HR in each period showed no significant difference between SSG_S_ and SSG_L_ (Figure 1). Mean HR was significantly lower in period 1 than in period 2 (Corrected-*p* < 0.05, ES = 0.85). Peak HR was significantly lower in period 1 than in period 2 (Corrected-*p* < 0.05, ES = 0.82) and period 3 (Corrected-*p* < 0.05, ES = 0.95). There was no significant difference in BLa or RPE between SSG_S_ and SSG_L_ (Table 1). RPE was significantly higher in period 4 than in period 1 (Corrected-*p* < 0.01, ES = 2.11), period 2 (Corrected-*p* < 0.01, ES = 1.32), and period 3 (Corrected-*p* < 0.01, ES = 0.61) (Table 1).

**Figure 2:**
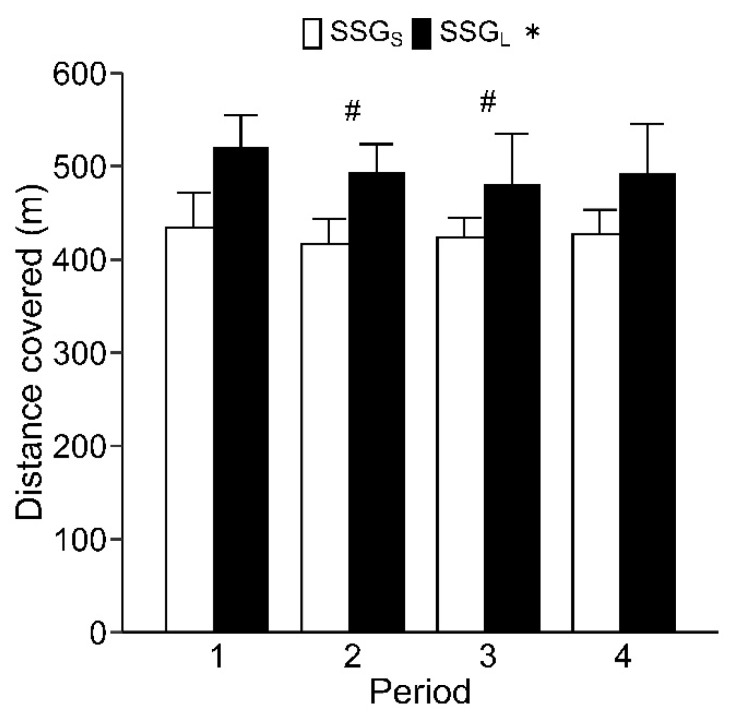
Distance covered during each period in SSG_S_ and SSG_L_. * Significant difference (Corrected-*p* < 0.05) between SSG_S_ and SSG_L_. # Significant difference (Corrected-*p* < 0.05) compared with period 1. Values are presented as mean ± standard deviation.

The pitch size × period interaction was significant (*p* < 0.05) for distances covered during jogging and quick running, but not total distance covered; distances covered during walking, high-intensity running, and sprinting; or peak speed. Total distance per session was 1702 ± 80 m in SSG_S_ and 1984 ± 166 m in SSG_L_; the total distance covered was significantly longer in SSG_L_ than in SSG_S_ (Corrected-*p* < 0.01, ES = 2.17) (Figure 2). The distance covered was longer in period 1 than in period 2 (Corrected-*p* < 0.05, ES = 0.84) and period 3 (Corrected-*p* < 0.05, ES = 0.86) (Figure 2). The distance covered per session while walking was significantly longer in SSG_S_ than in SSG_L_ (Corrected-*p* < 0.05, ES = 1.94) (Table 2). The distance covered while walking was significantly shorter in period 1 than in period 3 (Corrected-*p* < 0.05, ES = 1.39). The distance covered while jogging was significantly longer in SSG_S_ than in SSG_L_ in period 4 (Corrected-*p* < 0.01, ES = 1.87) and per full session (Corrected-*p* < 0.05, ES = 1.05) (Table 2). The distance covered during quick running was significantly longer in SSG_L_ than in SSG_S_ in period 1 (Corrected-*p* < 0.01, ES = 2.20), period 2 (Corrected-*p* < 0.01, ES = 1.33), and period 4 (Corrected-*p* < 0.05, ES = 1.12) and per full session (Corrected-*p* < 0.01, ES = 1.75). The distance covered per session during high-intensity running was significantly longer in SSG_L_ than in SSG_S_ (Corrected-*p* < 0.01, ES = 2.45) (Table 2). There was no significant difference between SSG_S_ and SSG_L_ in the distance covered while sprinting (Table 2). The peak speed was significantly higher in SSG_L_ (22.7 ± 2.4 km/h) than in SSG_S_ (22.0 ± 1.7 km/h) (Corrected-*p* < 0.05, ES = 1.35).

**Figure 3:**
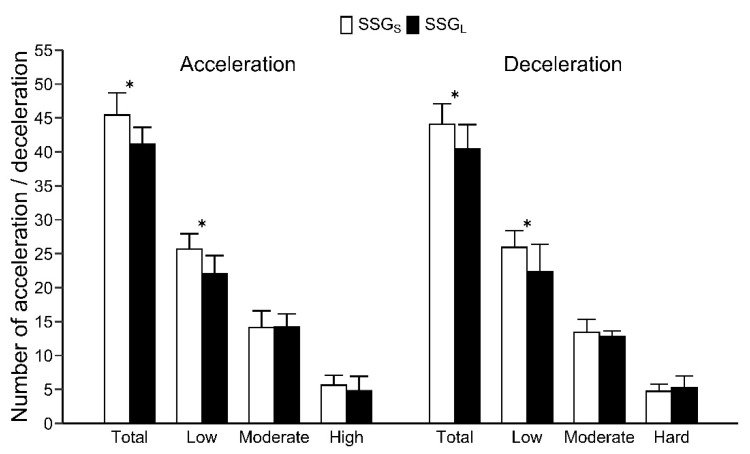
Accelerations and decelerations per period in SSG_S_ and SSG_L_. * Significant difference (Corrected-*p* < 0.05) between SSG_S_ and SSG_L_. Values are presented as mean ± standard deviation.

The pitch size × period interaction was not significant for the number of accelerations or decelerations in any category. The number of accelerations and decelerations, calculated as the mean per period, are shown in Figure 3. The number of accelerations in all categories (SSG_S_: 45 ± 3, SSG_L_: 41 ± 3, Corrected-*p* < 0.01, ES = 1.48) and low (SSG_S_: 26 ± 2, SSG_L_: 22 ± 3, Corrected-*p* < 0.01, ES = 1.45), and the number of decelerations in all categories (SSG_S_: 44 ± 3, SSG_L_: 40 ± 4, Corrected-*p* < 0.05, ES = 1.10) and low (SSG_S_: 26 ± 2, SSG_L_: 22 ± 4, Corrected-*p* < 0.05, ES = 1.07), were significantly greater in SSG_S_ than in SSG_L_ sessions (Figure 3). The number of decelerations (all categories) was significantly greater in period 1 than in period 2 (Corrected-*p* < 0.01, ES = 1.43). The number of high accelerations (SSG_S_: 6 ± 1, SSG_L_: 5 ± 2) and moderate accelerations (SSG_S_: 14 ± 2, SSG_L_: 14 ± 2), and the number of hard decelerations (SSG_S_: 5 ± 1, SSG_L_: 5 ± 2) and moderate decelerations (SSG_S_: 13 ± 2, SSG_L_: 13 ± 1) were not significantly different between SSG_S_ and SSG_L_ (Figure 3).

The pitch size × period interaction was not significant for number of passes, percentage of successful passes, number of catching errors, number of turnovers, or number of interceptions. The number of passes, percentage of successful passes, and number of interceptions were not significantly different between SSG_S_ and SSG_L_ (Table 3). The number of catching errors per session (Corrected-*p* < 0.05, ES = 0.95) and turnovers per session (Corrected-*p* < 0.05, ES = 1.24) were significantly greater in SSG_S_ than in SSG_L_ (Table 3). There was no significant difference in technical demands (number of actions and errors) between periods (Table 3).

## 4. Discussion

The present study investigated the physical, physiological, and technical demands on players in ultimate Frisbee SSG using two pitches of different sizes (SSG_S_: 30 × 15 m, SSG_L_: 40 × 20 m). Mean HR, peak HR, and BLa were similar between SSG_S_ and SSG_L_ (*p* > 0.05). The total distance covered and the distance covered during quick running and high-intensity running were significantly longer in SSG_L_ than in SSG_S_ (*p* < 0.05). The number of accelerations, decelerations, catching errors, and turnovers were significantly greater in SSG_S_ than in SSG_L_ (*p* < 0.05). To the best of the authors’ knowledge, this is the first study to compare exercise intensity in ultimate Frisbee SSG using two different pitch sizes.

Although HR responses were similar when playing SSG on both small and large pitches (Figure 1), and regular ultimate Frisbee games [1,2,4], SSG elicited higher BLa (all sessions: 11.6 ± 4.7 mmol/L) and longer distance covered per minute (SSG_S_: 106 ± 5 m/min, SSG_L_: 124 ± 10 m/min) than those elicited in regular games [1,2,4]. SSG may be able to elicit greater intensity activity than regular games because the frequency of participation in attacks is increased. Additionally, players may have been able to work at high intensities because the duration of SSG periods was short (4 min) and the breaks (5 min) between periods were sufficiently long. Madueno et al. [1] reported that mean HR, peak HR, and BLa during a 36 min game involving recreational male ultimate Frisbee players were 172 ± 12 bpm, 190 ± 14 bpm, and 5.5 ± 1.6 mmol/L, respectively. Krustrup and Mohr [4] reported that the total distance covered by competitive male ultimate Frisbee players was 4.70 ± 0.47 km (87 m/min) during a 54 min game. Players presumably maintain high exercise intensity during ultimate Frisbee by increasing the utilization of the anaerobic glycolytic energy system, given that BLa measured after play [1,2] exceeds the common anaerobic threshold (4.0 mmol/L) [21,22,23]. Therefore, high-intensity training that stimulates the glycolytic pathway seems to be especially important for ultimate Frisbee players to improve intramuscular buffering and lactate oxidation capabilities. Our study suggests that ultimate Frisbee SSG is particularly effective in stimulating the glycolytic pathway, as it can elicit a higher BLa than those elicited in regular games (4.3–8.4 mmol/L) [1,2], as well as SSGs of other sports (2.2–9.6 mmol/L) [8].

The present study showed that the total distance covered, distances covered during quick running and high-intensity running, and peak speed were significantly greater in SSG_L_ (40 × 20 m) than in SSG_S_ (30 × 15 m) (Table 2). In SSG_L_, the large playing area allowed for longer disc-throw distances, and thus the receivers may have covered longer distances at faster running speeds to catch the disc. Likewise, many previous studies have shown that distances covered increases with larger SSG pitch sizes in various sports [10,17,24,25,26,27]. For example, one study showed that the total distance covered (1326 ± 13 m vs. 957 ± 24 m) and distances covered with moderate velocity (3–5 m/s, 616 ± 16 m vs. 296 ± 15 m) and high velocity (5–7 m/s, 187 ± 11 m vs. 93 ± 7 m) were longer on a large pitch (70 × 40 m) than on a small pitch (40 × 10 m) in an 8 min rugby SSG [10]. The use of SSGs with a large pitch size can effectively lengthen the distance covered and increase speeds for team sports players.

However, HR, BLa, and RPE were not significantly different between SSG_L_ and SSG_S_ (Figure 1, Table 1). These variables may have been affected by the higher number of accelerations and decelerations in SSG_S_ than in SSG_L_ (Figure 3); not only speeds (or distance covered), but also acceleration and deceleration determine metabolic load [28]. The physiological demands of SSG_L_ associated with increased distances were equivalent to the physiological demands in SSG_S_, associated with more instances of acceleration/deceleration. In SSG_S_, players may have used frequent acceleration, deceleration, and changes in direction to increase the attacking space in a limited space. Additionally, the SSG_S_ seems to have led to increased technical errors (e.g., catching errors and turnovers) because of spatial constraints caused by reducing the space to attack [29,30]. Our study suggests that the use of SSG with a small pitch size enhances agility and technical difficulty. 

In this study, mean and peak HR were significantly lower in period 1 than in period 2 (Figure 1), and distance covered was longer in period 1 than in periods 2 and 3 (Figure 2). Previous studies have also shown a gradual increase in HR and gradual decrease in distance covered with repeated bouts of SSG [12,13]. These findings suggest that cardiovascular load gradually increased with repeated bouts of SSGs. Additionally, previous studies have shown that repeated bouts of soccer SSGs reduces technical actions and increases technical errors [12,13], which is likely a consequence of fatigue. However, the numbers of technical actions and errors did not change from period 1 to period 4 in our study (Table 3). Accumulated fatigue in ultimate Frisbee, in which the upper arm is used, may have less influence on technical actions than in soccer, in which technical actions are performed with the feet. 

Ultimate Frisbee players can be divided into two playing positions (cutters and handlers) [31]. Cutters mainly conquer the end zone of opposing teams, and handlers facilitate movement of the disc across the pitch. During the match, as cutters must effectively create space between opposing players to successfully receive passes, they perform more intense acceleration and deceleration than handlers [31]. As the effect of SSG training may differ between cutters and handlers, future studies should investigate the relationship between playing position and the effects of SSG training. Furthermore, it is necessary to consider the appropriate SSG training method for each playing position.

This study has some limitations. The sample size in this study (*n* = 9) was small, which may preclude generalization of these results. We did not measure the fitness level of each player, and the maximum HR of each player was not determined using an incremental treadmill test or field test, as it was estimated based on age. Each player performed only one SSG_S_ and one SSG_L_ session. As the physiological, physical, and technical demands during SSG might be affected by several factors, such as the wind, more than two sessions would be preferable to improve the reliability of the data. Furthermore, as we conducted only two trials (SSG_S_ and SSG_L_), future studies should systematically examine the influences of pitch size on the exercise intensity during SSG with increased numbers of pitch sizes (by using more than three trials).

## 5. Conclusions

The present study investigated the physical, physiological, and technical demands on players in ultimate Frisbee SSG (four, 4 min periods) using two pitches of different size (SSG_S_: 30 × 15 m; SSG_L_: 40 × 20 m). Ultimate Frisbee SSG elicited a high BLa (all sessions: 11.6 ± 4.7 mmol/L). In terms of the influence of pitch size, the total distance covered, and the distances covered during quick running and high-intensity running were significantly greater in SSG_L_ than in SSG_S_ (*p* < 0.05). On the other hand, the number of accelerations, decelerations, catching errors, and turnovers were significantly greater in SSG_S_ than in SSG_L_ (*p* < 0.05). With repeated bouts of SSGs, HR gradually increased and the distance covered gradually decreased, but the number of technical actions and errors remained fairly constant. This study suggests that ultimate Frisbee SSGs provide high-intensity training that stimulates the glycolytic pathway; therefore, manipulating the pitch size is effective to modulate the physical demands of SSG.

## Figures and Tables

**Table 1:** Blood lactate concentration and rating of perceived exertion during each period in SSG_S_ and SSG_L_.

		Period 1	Period 2	Period 3	Period 4	Mean
SSG_S_	BLa (mmol/L)	11.2 ± 3.8	11.4 ± 4.4	11.6 ± 5.0	11.2 ± 6.1	11.3 ± 4.7
	RPE	14.6 ± 1.0	14.9 ± 1.8	15.7 ± 2.0	16.7 ± 1.8 $	15.4 ± 1.8
SSG_L_	BLa (mmol/L)	11.1 ± 4.9	11.8 ± 4.0	12.1 ± 4.7	12.1 ± 5.6	11.8 ± 4.6
	RPE	14.0 ± 1.2	14.9 ± 1.4	15.9 ± 1.9	17.0 ± 1.6 $	15.4 ± 1.9

$ Significant difference (Corrected-*p* < 0.05) compared with other periods. Values are presented as mean ± standard deviation. BLa: blood lactate concentration (mmol/L) (period 1: *n* = 6, period 3: *n* = 8). RPE: rating of perceived exertion. SSG_S_: small SSG. SSG_L_: large SSG.

**Table 2:** Distance covered in five speed categories during each period in SSG_S_ and SSG_L_.

		Period 1	Period 2	Period 3	Period 4	Total
SSG_S_	Walking (m)	55 ± 9	58 ± 5	60 ± 6 #	56 ± 9	230 ± 15
	Jogging (m)	129 ± 17	129 ± 13	124 ± 10	133 ± 15	515 ± 34
	Quick running (m)	171 ± 25	175 ± 22	173 ± 18	178 ± 26	696 ± 69
	High-intensity running (m)	77 ± 21	53 ± 18	67 ± 18	58 ± 20	255 ± 44
	Sprinting (m)	2 ± 3	1 ± 3	1 ± 2	1 ± 3	5 ± 5
SSG_L_	Walking (m)	38 ± 6	49 ± 9	47 ± 11 #	48 ± 10	182 ± 32 *
	Jogging (m)	124 ± 11	114 ± 19	126 ± 19	106 ± 14 †	470 ± 49 *
	Quick running (m)	240 ± 36 †	208 ± 27 †	199 ± 34	212 ± 35 †	860 ± 112 *
	High-intensity running (m)	112 ± 26	113 ± 22	98 ± 35	116 ± 36	439 ± 95 *
	Sprinting (m)	6 ± 12	8 ± 7	9 ± 9	8 ± 13	32 ± 33

* Significant difference (Corrected-*p* < 0.05) compared with SSG_S_. † Significant difference (Corrected-*p* < 0.05) compared with the same period of SSG_S_. # Significant difference (Corrected-*p* < 0.05) compared with period 1. Values are presented as mean ± standard deviation. SSG_S_: small SSG. SSG_L_: large SSG.

**Table 3:** Technical demands of each period in SSG_S_ and SSG_L_.

		Period 1	Period 2	Period 3	Period 4	Total
SSG_S_	Passes	7 ± 3	7 ± 3	7 ± 3	7 ± 3	27 ± 10
	Percentage of successful passes (%)	75 ± 18	83 ± 20	74 ± 21	79 ± 17	78 ± 18
	Catching errors	0 ± 0	0 ± 1	1 ± 1	1 ± 1	2 ± 1
	Turnovers	2 ± 1	2 ± 1	3 ± 2	2 ± 1	8 ± 2
	Intercepts	1 ± 1	0 ± 1	0 ± 1	0 ± 1	2 ± 1
SSG_L_	Passes	8 ± 2	7 ± 2	7 ± 3	6 ± 2	28 ± 7
	Percentage of successful passes (%)	77 ± 10	85 ± 14	87 ± 13	85 ± 13	84 ±13
	Catching errors	0 ± 0	0 ± 0	0 ± 0	0 ± 0	1 ± 1 *
	Turnovers	2 ± 1	1 ± 1	1 ± 1	1 ± 1	6 ± 2 *
	Intercepts	0 ± 0	0 ± 1	1 ± 1	0 ± 0	2 ± 1

* Significant difference (Corrected-*p* < 0.05) compared with SSG_S_. Values are presented as mean ± standard deviation. SSG_S_: small SSG. SSG_L_: large SSG.

## Data Availability

Data are available from the authors, upon reasonable request.
